# An Analytic Approach Using Candidate Gene Selection and Logic Forest to Identify Gene by Environment Interactions (G × E) for Systemic Lupus Erythematosus in African Americans

**DOI:** 10.3390/genes9100496

**Published:** 2018-10-15

**Authors:** Bethany J. Wolf, Paula S. Ramos, J. Madison Hyer, Viswanathan Ramakrishnan, Gary S. Gilkeson, Gary Hardiman, Paul J. Nietert, Diane L. Kamen

**Affiliations:** 1 Department of Public Health Sciences, Medical University of South Carolina, Charleston, SC 29425, USA; ramosp@musc.edu (P.S.R.); madison.hyer@osumc.edu (J.M.H.); ramakris@musc.edu (V.R.); hardiman@musc.edu (G.H.); nieterpj@musc.edu (P.J.N.); 2 Division of Rheumatology and Immunology, Department of Medicine, Medical Univeristy of South Carolina, Charleston, SC 29425, USA; gilkeson@musc.edu (G.S.G.); kamend@musc.edu (D.L.K.); 3 Center for Genomic Medicine, Department of Medicine, Medical Univeristy of South Carolina, Charleston, SC 29425, USA; 4 Division of Nephrology, Department of Medicine, Medical Univeristy of South Carolina, Charleston, SC 29425, USA; 5 School of Biological Sciences & Institute for Global Food Security, Queens University Belfast, Stranmillis Road, Belfast BT9 5AG, UK

**Keywords:** candidate genes, gene–environment interactions, logic forest, systemic lupus erythematosus

## Abstract

Development and progression of many human diseases, such as systemic lupus erythematosus (SLE), are hypothesized to result from interactions between genetic and environmental factors. Current approaches to identify and evaluate interactions are limited, most often focusing on main effects and two-way interactions. While higher order interactions associated with disease are documented, they are difficult to detect since expanding the search space to all possible interactions of *p* predictors means evaluating 2^*p*^ − 1 terms. For example, data with 150 candidate predictors requires considering over 10^45^ main effects and interactions. In this study, we present an analytical approach involving selection of candidate single nucleotide polymorphisms (SNPs) and environmental and/or clinical factors and use of Logic Forest to identify predictors of disease, including higher order interactions, followed by confirmation of the association between those predictors and interactions identified with disease outcome using logistic regression. We applied this approach to a study investigating whether smoking and/or secondhand smoke exposure interacts with candidate SNPs resulting in elevated risk of SLE. The approach identified both genetic and environmental risk factors, with evidence suggesting potential interactions between exposure to secondhand smoke as a child and genetic variation in the *ITGAM* gene associated with increased risk of SLE.

## 1. Introduction

Many complex human diseases have been hypothesized to be the result of interactions between genetic and environmental risk factors [1,2,3,4,5,6,7,8,9]. Research studies aimed at detecting potential gene by environment (G × E) interactions as risk factors for human disease most often take one of two approaches. The first approach, often applied in genome-wide association studies, evaluates all two-way interactions. However, higher order interactions would not be detected using the this approach since expanding the search space to include higher order interactions is prohibitively laborious and computationally intensive, as evaluating all possible main effects and interactions in a data set with *p* predictors would mean evaluating $2^{p}-1$ terms [9,10]. A second approach is to identify a set of candidate factors and/or interactions between these factors. The selection of the “best” subset of genetic and environmental factors may be based on the marginal effects of each factor passing a specific statistical significance threshold. In this case, only those factors that have a strong marginal effect are selected for interaction screening, which will fail to identify those factors with minimal marginal effects but strong interaction effects [2,8,9]. Alternatively, a subset of candidate genetic and environmental factors may be selected *a priori* [10]. Selecting candidate single nucleotide polymorphisms (SNPs) from genome wide data coupled with the environmental exposures provides a sufficiently concise and targeted sample space to be thorough while computationally manageable.

Identification of candidate variants and exposures can be prioritized based on *a priori* knowledge (e.g., reported association, biomedical data from databases, involvement in relevant biological mechanisms or pathways) and can be facilitated through existing literature and databases. If a suitable subset of candidate genes and environmental exposures can be identified, the analytical approach to evaluate the possible interactions among these factors must be considered. Statistical approaches such as case-only studies have been proposed to improve the efficiency of interaction identification in such studies [11,12,13]. However, results from such designs may be misleading as there is an assumption of independence between factors, which if violated can lead to erroneous conclusions [12,14,15]. Additionally, such studies typically focus on two-way interactions as each interaction is evaluated individually, which can be a limitation if seeking to identify interactions with more than two terms [9,10,14,16]. For example, data with only 25 predictors still requires evaluating over 10^7^ terms (predictors) while data with 150 predictors would require evaluating over 10^45^ terms. Machine learning methods such as artificial neural networks, support vector machines, and forest approaches offer flexibility in modeling outcomes and can incorporate complex relationships such as higher order interactions in modeling disease outcomes based on a large number of predictors [17,18,19,20,21,22]. However, analytic approaches should provide guidance for determining the subset of predictors and predictor interactions from among a larger set that are most relevant for determining outcome. Both random forest and Logic Forest provide quantitative importance measures for individual predictors allowing them to be ranked according to their relative importance in determining an outcome [17,22]. However, predictor importance for each variable represents the marginal effect of a predictor and if a set of predictors is associated with the outcome only through interactions effects, these marginal importance measures may mask such interaction effects [23]. Unlike random forest, Logic Forest also provides a quantitative measure of importance for interactions identified by the forest, which is advantageous in complex disease settings where interactions among genetic and environmental factors rather than main effects lead to disease. Despite the availability and usefulness of such tools, they have been under utilized. An ideal approach would combine identification of candidate factors based on prior knowledge with an efficient method for evaluating the space of possible interactions, including higher order interactions, among these candidate factors.

In this paper, we present an analytic approach to evaluate main effects and interactions between genetic and environmental factors associated with a disease outcome by coupling selection of relevant genetic and environmental factors based on available literature and public databases with a machine learning approach, Logic Forest. To illustrate this approach, we examine varying degrees of tobacco smoke exposure as environmental factors, disease-associated SNPs as genetic factors, and their individual and combined associations with the diagnosis of systemic lupus erythematosus (SLE) in a cohort from the Sea Island Gullah population of South Carolina. The Gullah population is a distinctive group of African Americans from the coastal Sea Islands of South Carolina and Georgia who are descendants of enslaved Africans from the African Rice Coast [24]. On many plantations, Africans vastly outnumbered Europeans, and the Gullah remained in the geographically isolated Sea Islands until recent times [24,25,26]. This population is unique in that they have low non-African genetic admixture [25,26] and high ancestral homogeneity from their ancestral home, Sierra Leone [27,28,29], offering a unique opportunity to study genetic and environmental disease risk factors. SLE is a “prototype” autoimmune rheumatic disease with a well substantiated genetic etiology and many of the SNPs identified as increasing the risk for SLE are in genes that enhance immune reactivity [30,31,32,33,34,35,36,37,38]. Additionally, given that the concordance rate between monozygotic twins only ranges between 24% and 35% [31], epigenetic or environmental factors are likely to have an important role in SLE susceptibility. Known environmental triggers in SLE include ultraviolet (UV) light, silica dust, certain infections, and smoking [39]. We apply our proposed approach to evaluate associations between risk of SLE with genetic factors thought to amplify the inflammatory/immune response to tobacco smoke exposure, which has been implicated in earlier research [40]. Results of the analysis found evidence of both a main effect for smoke exposure and several interactions between genetic factors and smoke exposure, demonstrating the applicability of our approach.

## 2. Materials and Methods

**Figure 1:**
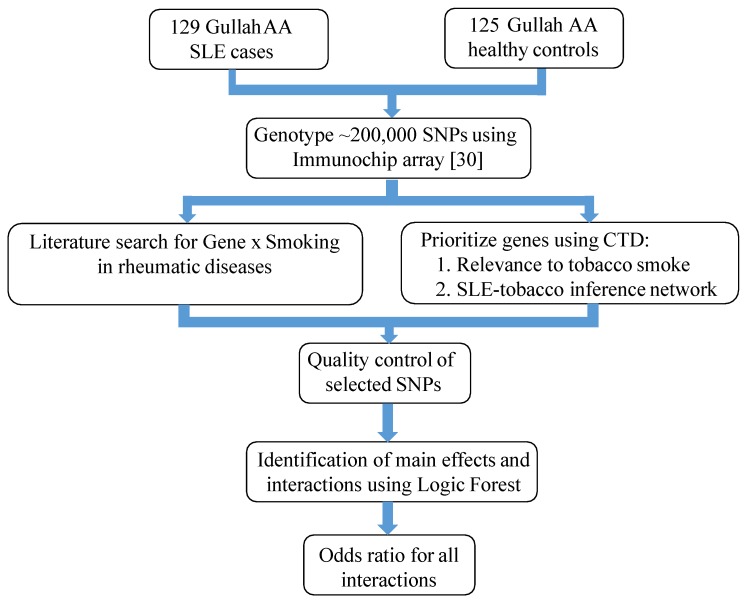
Flowchart of the proposed analytic approach. AA: African American; SLE: Systemic lupus erythematosus; CTD: Comparative Toxicogenomics Database, and SNP: Single nucleotide polymorphism.

We present an analytical approach for identifying main effects and interactions between genetic and environmental factors associated with a disease outcome. The approach involves selection of candidate genetic and/or environmental factors, use of a machine learning algorithm to identify important main effects and interactions in disease, followed by confirmation of the association between interactions identified by the algorithm using logistic regression. To give this theoretical approach context, it is applied to a study examining the association between SNPs and cigarette smoke exposure with risk of developing SLE as shown in Figure 1.

### 2.1. Study Subjects and Design

The Gullah population is a distinctive group of African Americans from the coastal Sea Islands of South Carolina and Georgia they are descendants of enslaved Africans from the African Rice Coast [24] and thus represent a unique population of African Americans, which, while not a genetic isolate, is a more genetically homogeneous group relative to other African Americans [25,26,27,28,29,41]. Systemic lupus erythematosus is also known to have a high disease load in African Americans relative to Americans of European descent with an estimated prevalence in South Carolina of $\frac{1}{200}$ in African American women; the prevalence in the Gullah is unknown, but it is believed to be similar [41].

The SLE study used a case control design, and subjects were selected from people participating in the SLE in Gullah Health (SLEIGH) Study, which began recruitment in 2003 [42]. Systemic lupus erythematosus cases fulfilled the 1997 American College of Rheumatology classification criteria for “definite” SLE [43]. Race was self-reported and Gullah ancestry was self-identified as African American (AA) Gullah from the Sea Island region of South Carolina, with all known grandparents being of Gullah descent [42,44,45]. Unrelated non-SLE Gullah controls were also recruited by asking the cases to “bring a friend” of the same gender and community to the screening visit. As described in our recent manuscript [45], first-degree relatives were not considered for the analysis. These subjects received a clinical examination by a rheumatologist to ensure they did not meet criteria for any inflammatory rheumatologic disease before inclusion in the genetic studies as unaffected Gullah controls. This study was approved by the Medical University of South Carolina Institutional Review Board (Pro#00021985, approved 1/15/2013). All study participants provided written consent prior to study enrollment.

Genotypic data was available on 129 Gullah AA SLE cases and 125 AA unrelated controls genotyped on the Immunochip genotyping array [45]. Tobacco smoke exposure, including both secondhand smoke exposure as a child and current smoking status, was collected as a part of the SLEIGH study protocol. At baseline, each subject was asked the following questions as part of an in-person interview related to smoking: “Have you ever smoked cigarettes?” (If yes) “What was the maximum daily amount (packs per day) smoked?” “What is the total number of years you smoked?” “Are you currently smoking?” “If not, how many years since quitting?”. Participants were also asked the following questions about secondhand smoke exposure: “Were you ever routinely exposed to passive smoke as an adult (at work or in the home)?” “Were you ever exposed to passive smoke as a child (before age 18)?”. From responses to these questions, four binary variables were created for each case and control to indicate whether or not they (1) had ever been a smoker prior to SLE diagnosis (for cases) or prior to their study visit (for controls), (2) were current smokers at the time of SLE diagnosis (for cases) or at their baseline visit (for controls), (3) were ever regularly exposed to secondhand smoke, and (4) were ever regularly exposed to secondhand smoke as a child (<18 years old). Twenty participants were missing information on smoking and smoke exposure data and were excluded for analysis.

### 2.2. Prioritization of SNPs

#### 2.2.1. Gene Selection

We searched the literature for reports of interactions between genetic variation and tobacco smoke in SLE and related rheumatic diseases. We identified genes with reported interactions with tobacco smoke in SLE (*NAT2*) [40] and rheumatoid arthritis (*HLA-DRB1* shared epitope [46], *PTPN22* [47], and *HMOX1* [48]). In addition to these candidate genes from the literature, we also used information compiled in the Comparative Toxicogenomics Database (CTD) [49], a database that contains curated scientific data describing relationships between chemicals/drugs, genes/proteins, diseases, phenotypes, pathways, and interaction modules. We used the CTD to prioritize genes relevant to tobacco smoke (*APOE, NFE2L2, IL6* and *CXCL8*) and genes in an inference network between tobacco smoke and SLE (*IRF5, ITGAM* and *ITGAX*; *IL6* is also part of this network).

#### 2.2.2. Genotypic Dataset and Quality Control

Genotypic data on 129 Gullah AA SLE cases and 125 AA controls genotyped on the Immunochip array was subject to the following quality control (QC) filters: exclusion of individuals with missing genotypes, markers that did not statistically conform to Hardy–Weinberg Equilibrium (HWE) (at p<0.001) in controls, markers with missing data, and markers with minor allele frequency (MAF) < 0.05. We used all the SNPs that met these QC thresholds in a region including ±5 kb around each gene. Most promoters are located within 1 kb of the transcription start site, a 5 kb flanking region around a gene is a common and reasonable choice. For the four genes with previously reported interactions with tobacco smoke (*NAT2, HLA-DRB1, PTPN22* and *HMOX*), we searched the 1000 Genomes and HapMap Projects for SNPs that tag the reported alleles (as defined by an r-squared > 0.4 in the YRI (Yoruba in Ibadan, Nigeria) population) that might have been genotyped and met QC in our dataset. Populations of African ancestry have decreased linkage disequilibrium (LD) and a rapid decay of LD with distance genome-wide relative to populations of European ancestry [45]. A threshold of r-squared > 0.4 is thus reasonable to identify proxy SNPs in our population. Finally, the genotypic cluster plots for each SNP were visually inspected, and SNPs with poor or questionable plots (without clear cluster separation) were excluded. After applying these QC filters, the following were available for further analyses: *NAT2* (4 SNPs), *HLA-DRB1* (6 SNPs), *APOE* (2 SNPs), *IL6* (17 SNPs), *CXCL8* (1 SNP), *IRF5* (20 SNPs), *ITGAM* (67 SNPs), and *ITGAX* (31 SNPs). Genotype frequencies for each of the SNPs discussed in the manuscript are listed in Supplemental Table S1. Thirty participants failed to meet quality control parameters and were excluded from the analysis.

### 2.3. Identification of Important Main Effects and Interactions

The primary goal of the SLE study was to identify potential gene×gene and gene×environment interactions associated with risk of SLE among the Gullah population. We used a binary classification algorithm to identify main effects and interactions among the candidate SNPs and smoke exposure for classifying individuals according to SLE status.

#### 2.3.1. Logic Forest

**Figure 2:**
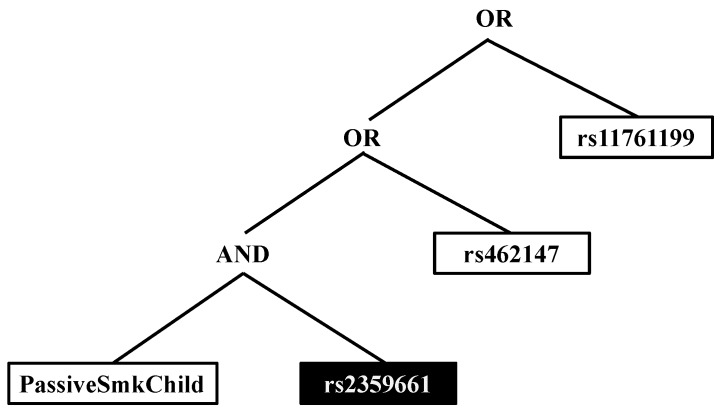
Example of a logic regression tree. White boxes represent the predictor, in the case of SNPs, the recessive effect of the minor allele, and black boxes represent the complement of that predictor (e.g., for a SNP, this means the dominant effect of the major allele). There are three independent predictors/predictor interactions identified within the tree: (1) exposure to passive smoking as a child and having at least one copy of the major allele of rs2359661 (A) in *ITGAM*; (2) having two copies of the minor allele of rs4632147 (T) in *ITGAX*; and (3) having two copies of the minor allele of rs11761199 (G) in *IRF5*.

Logic Forest (LF) is a machine learning algorithm designed to identify interactions among binary variables (for example, SNPs or smoking status) and quantify the importance of potential predictors and predictor interactions identified in the forest in terms of correctly classifying disease status [22]. Logic Forest does not require *a priori* specification of interactions as it iteratively evaluates the space of all possible interactions to identify the subset of interactions best able to classify disease status. The LF algorithm and methods for calculating LF model misclassification rate and predictor interaction importance have been previously described by [22] and detailed description of the algorithm can be found there. For completeness, we provide details of the algorithm here. Given data W={X,y} where $X={\left(x_{1},x_{2},\ldots ,x_{p}\right)}^{\prime }$ is an n×p matrix of binary predictors and $y={\left(y_{1},y_{2},\ldots ,y_{n}\right)}^{\prime }$ is a binary vector indicating disease status for i=1,2,…,n subjects, an LF model consists of a collection of *B* logic regression trees constructed from *B* bootstrap samples from data W and is denoted as $LF\left(W,B\right)=\left\{T^{1},T^{2},\ldots ,T^{B}\right\}=\left\{T^{b}\right\}$. A single logic regression tree, $T^{b}$, represents the predictors and predictor interactions, referred to as “prime implicants”, identified for the *b*-th bootstrap sample as being associated with having SLE. Trees in an LF model are allowed to grow up to maximum size of eight leaves. Thus, trees in the forest can explore interactions of up to eight variables. Figure 2 shows an example logic regression tree with three prime implicants identified as associated with SLE: (1) exposure to passive smoking as a child and having at least one copy of the major allele of rs2359661 (A) in *ITGAM*; (2) having two copies of the minor allele of rs4632147 (T) in *ITGAX*; and (3) having two copies of the minor allele of rs11761199 (G) in *IRF5*. When all predictor variables are categorical (e.g., SNPs), an interaction between two variables occurs when specific conditions for both variables must be met to confer additional risk of disease. For example, the first prime implicant for Figure 2 suggests that additional risk for SLE from having at least one copy of the major allele of rs4632147 occurs only if the subject also had passive smoke exposure as a child. For tree $T_{b}$ in the forest, subjects are predicted to have disease if they meet any of the conditions defined by the tree.

Predictions for the LF model of *B* trees is determined by the proportion of trees that predict the subject to have SLE. Each tree $T^{b}$ in the LF has an associated out-of-bag (OOB) dataset, $OOB\left(T^{b}\right)$, comprised of those observations left out of the *b*-th bootstrap sample that can be used for an unbiased estimate of the model’s prediction error (similar to internal bootstrap validation). The LF OOB prediction for observation $y_{i}$ is determined by Equation (1) where $I\left(W_{i}\in OOB\left(T^{b}\right)\right)$ is the indicator of the *i*-th observations membership in $OOB\left(T^{b}\right)$.

(1) $$\hat{y_{i}^{OOB}}\left(\left\{T^{b}\right\},x_{i}\right)=\left\{\begin{array}{cc} 1, & \mathrm{if}\frac{\sum_{b=1}^{B}\hat{y_{i}}\left(T^{b},x_{i}\right)I\left(W_{i}\in OOB\left(T^{b}\right)\right)}{\sum_{b=1}^{B}I\left(W_{i}\in OOB\left(T^{b}\right)\right)}\geq 0.5,\\ 0, & \mathrm{otherwise}. \end{array}\right.$$

Accordingly, the LF OOB misclassification rate is

(2) $$MC^{OOB}\left(\{T^{b}\},y,X\right)=\frac{1}{n}\sum_{i=1}^{n}{\left(y_{i}-\hat{y_{i}^{OOB}}\left(\left\{T^{b}\right\},x_{i}\right)\right)}^{2}.$$

Logic Forest also provides two quantitative measures of importance for all prime implicants identified in the forest. The first measure evaluates the change in classification error for each tree in the forest before and after permutation of the data. The misclassification rate for tree $T^{b}$ is

(3) $$MC^{OOB}\left(T^{b},y,X\right)=\frac{\sum_{i=1}^{n}{\left(y_{i}-\hat{y_{i}^{OOB}}\left(T^{b},x_{i}\right)\right)}^{2}I\left(W_{i}\in OOB\left(T^{b}\right)\right)}{\sum_{i=1}^{n}I\left(W_{i}\in OOB\left(T^{b}\right)\right)}.$$

Let $X^{\left(j\right)}$ be the matrix of predictors with $X_{j}$ randomly permuted, where $X_{j}$ can be an individual predictor or more generally a prime implicant. The importance of prime implicant $X_{j}$ is

(4) $$VI_{1}\left(X_{j}\right)=\frac{1}{B}\sum_{b=1}^{B}\left[MC^{OOB}\left(T^{b},y,X^{\left(j\right)}\right)-MC^{OOB}\left(T^{b},y,X\right)\right].$$

Values for Equation (4) range from –1 to 1 with positive values indicating a positive association between response y and prime implicant $X_{j}$. The second measure of prime implicant importance is the frequency with which the prime implicant occurs across trees in the forest and can be calculated according to Equation (5)

(5) $$VI_{2}\left(X_{j}\right)=\frac{1}{B}\sum_{b=1}^{B}I\left(X_{j}\in T^{b}\right),$$

where $I\left(X_{j}\in T^{b}\right)$ is an indicator of prime implicant $X_{j}$’s inclusion in tree $T^{b}$. Permutation *p*-values for importance measures for each prime implicant $X_{j}$ can be calculated by randomly permuting the outcome many times and fitting LF models to the data with the permuted outcome. The permutation *p*-value is the proportion of times LF models fitted to data with the outcome permuted yield an importance score for prime implicant $X_{j}$ as large as or larger than the importance score from the original model.

For analysis of the SLE study, three LF models including 200 logic regression trees each were fit using (1) the recessive effect of the minor allele for each SNP (i.e., subjects have two copies of the minor allele); (2) the dominant effect of the minor allele for each SNP (i.e., subjects having at least one copy of the minor allele); and (3) the genotypic model with two indicators for of the number of copies of the minor allele (with 0 being a reference group). Demographic and environmental variables, namely gender, passive smoke exposure as a child, passive smoke exposure as an adult, and smoking status as an adult were also considered in each model. Permutation *p*-values for prime implicants identified by LF models were calculated based on 500 LF models fitted to the data with SLE case-control status randomly permuted. All analyses were conducted in R v. 3.2.5 using the *LogicForest* package [50,51].

#### 2.3.2. Validation of Main Effects and Interactions

To further validate the association between prime implicants identified by the LF and response *y*, logistic regression models were also constructed to estimate odds ratios associated with each risk factor (i.e., main effects and interactions) identified using the LF approach.

## 3. Results

Twenty subjects were missing information on childhood and/or adult smoke exposure and 30 additional subjects had missing genotype information, thus the final study population included 204 participants with both genetic and environmental exposure data available, 100 of whom were diagnosed with SLE. There was no notable difference in sex or case/control status between subjects included in the final population compared to those who were excluded (data not shown). Participants included in the study were on average four years older than participants that were excluded (*p* = 0.042). A majority of the study participants were female (85.8%), consistent with the historical gender distribution for the disease. Participant demographic characteristics for cases and unrelated controls are shown in Table 1.

**Figure 3:**
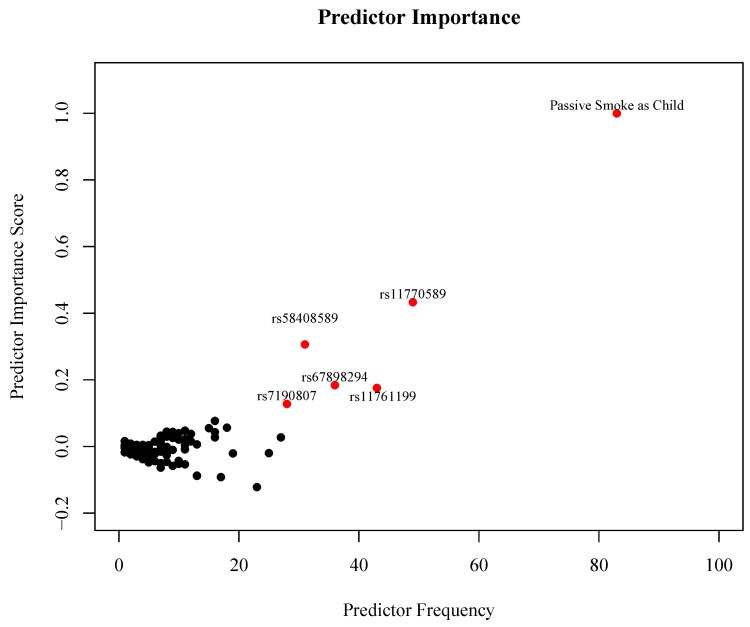
Predictor frequency by normalized predictor importance score for all predictors in the Logic Forest (LF) model. Points highlighted in red represent the predictors that have the largest combination of frequency and importance score.

The results from the LF model that included the recessive effect of the minor allele and the environmental and demographic variables are presented, since the gene–environment interactions identified in this model showed the strongest relationship with SLE status. Logic Forest identified 426 unique prime implicants across the 200 trees in the model. Figure 3 is a plot of the number of trees in the model that include each predictor by the normalized importance scores for each predictor. Points shown in red represent those predictors that have the largest combination of predictor frequency and importance score. As seen in Figure 3, the LF model identified passive smoke exposure as a child as the most important predictor of SLE status (permutation *p* < 0.01). The SNPs rs11770589 (*IRF5*), rs58408589 (*ITGAX*), rs67898294 (*ITGAX*), rs11761199 (*IRF5*), and rs7190807 (*ITGAM*) had both a high predictor importance score and occurred frequently in the LF model (permutation *p* < 0.01 for all).

**Figure 4:**
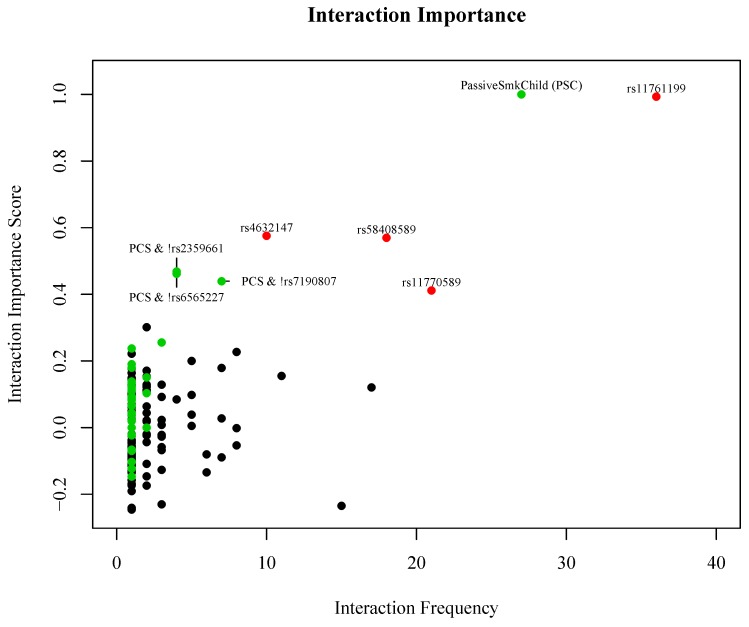
Interaction frequency by normalized interaction importance score for all interactions identified in the LF model. Points highlighted in red represent the interactions that have the largest combination of frequency and importance score. Points in green represent additional interaction terms identified in the forest that include passive smoke exposure as a child with at least one SNP.

Figure 4 shows the number of trees in the model that include each prime implicant by the normalized importance scores for all prime implicants that were identified in the forest. The most important and most frequent prime implicants identified in the forest were the main effects for passive smoke exposure as a child (permutation *p* = 0.008) and the following SNPs: rs4632147 (*ITGAX*), rs11761199 (*IRF5*), rs11770589 (*IRF5*), and rs58408589 (*ITGAX*) (permutation *p* = 0.006, 0.01, 0.01, and 0.028, respectively).

There are three additional interaction terms that were ranked as highly important and occurred with some regularity that included SNPs in the *ITGAM* gene and passive smoke exposure as a child (permutation *p* < 0.002 for all three interactions). The points in Figure 4 highlighted in red represent the interactions that have the largest combination of frequency and importance score. Points in green represent interaction terms identified in the forest that include passive smoke exposure as a child with at least one SNP. Passive smoke exposure as a child occurred in 88 of the 200 trees, and in 27 of those instances it occurred as a main effect. In the remaining 61 instances, it occurred as an interaction with different SNPs. Although the main goal of this analysis is to identify potential gene–gene and gene–environment interactions; for completeness, we also examined the ability of the LF model to discriminate SLE cases from controls. The estimated prediction error rate for the final LF model is 43%, with an area under the receiver operating characteristic (ROC) curve of 0.54 (ROC curve for the final model is shown in Supplemental Figure S1).

The Logic Forest model identified four main effects and three interactions as the most important predictors in for determining SLE status based on the importance score. Separate logistic regression models for these seven predictors that had the largest importance scores from the LF model were fit by including an indicator variable for whether or not the subject had the combination of exposures in the interaction. Table 2 shows the odds ratios and associated *p*-values for these logistic regression models. The LF model included indicators for the recessive effect of the minor allele; however, if the model found an interaction with the complement of a recessive effect, this is equivalent to the interaction term including at least one copy of the major allele (i.e., dominant effect of the major allele as noted in the last three interactions shown in Table 2). These results generally agree with the results from the LF model in that a majority of the prime implicants reported in the table have a statistically significant association with being SLE positive. The only exception is rs11770589 in the *IRF5* gene, which has a *p*-value from the logistic regression model of 0.18.

## 4. Discussion

In this study, we demonstrate the utility of the proposed analytical approach to examine main effects and interactions between 148 SNPs, gender, and four different types of smoke exposure in a well-characterized cohort of Gullah African Americans participating in the SLEIGH study. There are several key take-home points from the analysis of the SLE study. The LF model found strong evidence for an association between SLE status and passive smoke exposure as a child. Logic forest also consistently identified SNPs associated with SLE, including rs58408589, rs67898294, rs7190807, rs4632147, rs11770589, and rs11761199 (in the *IRF5, ITGAM*, and *ITGAX* genes). Finally, although passive smoke exposure as a child was clearly identified as a main effect (i.e., an independent risk factor), there was also evidence to suggest that it may also be involved in weak to moderate interactions with SNPs on the *ITGAM* gene (Table 2).

There are alternative statistical methods that one might consider for evaluating potential gene×gene or gene×environment interactions for SLE. For example, logistic regression is a traditional approach that could be used for such analyses. However, in order to evaluate the association between SLE and all potential two-way interactions involving the 153 predictors in our data set, one would need to examine 1532 =11,628 logistic regression models; potential three-way interactions would be even more cumbersome, as there would be almost 600,000 of them. Nonparametric decision tree methods are easily interpretable and have flexibility to identify interactions among predictors [52,53]. However, decision tree models may be unstable, in that small changes in the data can result in very different models [17,52,54,55]. Ensemble models, a collection of decision trees developed using bootstrap samples or weighted samples of a dataset improve model stability and prediction accuracy compared to single tree approaches [17,22,55,56,57,58]. Random forest (RF) and Logic Forest (LF) are ensemble extensions of two decision tree methods [17,22]. Both methods also provide a quantitative measure of the relative importance of predictors used in the model. However, LF has an additional advantage over RF in that it also has a quantitative importance measure for interactions found in the forest, rather than just individual predictors, making it ideal for identifying potential gene×gene and gene×environment interactions in SLE development.

Our findings from the SLE study are not the first to demonstrate that certain SNPs may interact with environmental exposures, such as smoking, in a way that increases the risk of developing SLE. In a Japanese cohort, investigators found significant evidence of increased risk of SLE associated with smoking, highest among those with polymorphisms in the *NAT2* gene influencing metabolic enzymes involved in reactive oxygen species production [40]. They identified a possible gene×environment interaction, where smokers with the slow acetylator genotype of *NAT2* were found to have a higher risk of SLE (Odds Ratio = 6.44, 95% CI = 3.07–13.52) when compared to non-smokers with the rapid acetylator genotype of *NAT2*. Our study was the first to find passive smoke exposure as a child (childhood exposure to secondhand smoke) to be a significant risk factor for SLE. The main effect of childhood smoke exposure and the interactions between several SNPs on the *ITGAM* gene were also significant in univariate logistic regression models of SLE status. Additionally, two SNPs on the *ITGAX* gene and two SNPs on the *IRF5* gene were also identified by the LF model, though only three of the four SNPs were also significant in subsequent logistic regression models. Logic Forest does not assume linearity in the logit link between predictors and outcome as logistic regression does, which may explain the discrepancies in significance of rs11770589 on the *ITGAX* gene.

Given the exploratory nature of these analyses and the limited sample size of our study population, replication would greatly improve the credibility of the associations identified in this study. Unfortunately, there are no large scale genetic studies of SLE (or of any related autoimmune disorder) in African Americans. Furthermore, the population selected for this study (Gullah African Americans) was chosen for their documented high genetic homogeneity [42,45] and a replication cohort of genetically similar individuals does not exist. Thus, the associations reported would need to be validated in a future study. Additional potential limitations of this study include recall bias and reliance on self-report to ascertain the individuals’ smoking and exposure status. These findings should be considered as part of the “discovery” or “hypothesis generating” process of understanding whether and how smoke exposure may interact with certain genes and should not be construed as definitive proof. A detailed understanding of the mechanisms underlying SLE pathogenesis will continue to require large databases of study subjects, with well-characterized environmental exposures and genetic information. Machine learning algorithms, such as Logic Forest, will inevitably be required to help sort through the ever expanding combination of potential risk factors for disease.

## 5. Conclusions

This study illustrates the utility of a novel approach to identify interactions between genetic and environmental risk factors for disease. The complexity of many human diseases, which likely result from interactions between genetic and environmental factors, emphasizes the importance of evaluating such interactions when examining disease etiology. The challenge for such studies is the number of possible interactions in data with even a modest number of individual predictors. For example, in the SLE study presented here, there are 2^153^ − 1 = 5.7 × 10^45^ possible interactions. The approach presented here combines candidate gene selection and a machine learning method for identification and quantification of the relative importance of interactions from among all possible interactions in determining disease state, followed by confirmation of the association between those predictors/interactions with disease outcome. Applying this approach to a study examining genetic and environmental factors in SLE identified childhood exposure to secondhand smoke (PSC) as an independent effect and interactions between PSC and SNPs on ITGAM, providing additional evidence that SLE is a disease with a complex etiology and is the first study to find childhood exposure to secondhand smoke to be a significant risk factor for SLE.

## Figures and Tables

**Table 1:** Participant characteristics by SLE status.

Characteristic	Control (*n* = 104)	SLE (*n* = 100)	*p*-Value *
Age (Mean ± Std Dev)	42.6 ± 11.7	38.6 ± 13.4	0.022
Female (*n*, %)	87 (83.6)	88 (88.0)	0.491
Passive Smoke Exposure as a Child (*n*, %)	28 (26.9)	41 (41.0)	0.048
Passive Smoke Exposure as an Adult (*n*, %)	18 (17.3)	20 (20.0)	0.754
Ever Smoker (*n*, %)	24 (23.1)	24 (24.0)	1.000
Current Smoker (*n*, %)	13 (12.5)	17 (17.0)	0.478

* *p*-values reported in the table for the association with SLE status are based on a two-sample *t*-test for age and chi-square test for all categorical variables.

**Table 2:** Odds ratios with 95% confidence intervals (CI) from a series of logistic regression models. The implied reference category for each odds ratio is the complement of the effect defined in the first column.

Effect	Gene	Odds Ratio (95% CI)	Unadjusted *p*-Value
Passive Smoke Exposure as Child (PSC)		1.88 (1.01, 3.55)	0.039
2 copies of the minor allele of rs4632147 (T)	*ITGAX*	3.09 (1.09, 10.1)	0.023
2 copies of the minor allele of rs58408589 (C)	*ITGAX*	2.96 (1.23, 7.75)	0.011
2 copies of the minor allele of rs11761199 (G)	*IRF5*	7.69 (1.01, 352)	0.033
2 copies of the minor allele of rs11770589 (A)	*IRF5*	1.65 (0.81, 3.42)	0.179
PSC & >1 copy of the major allele of rs2359661 (A)	*ITGAM*	2.28 (1.18, 4.48)	0.009
PSC & >1 copy of the major allele of rs7190807 (G)	*ITGAM*	2.46 (1.25, 4.92)	0.005
PSC & >1 copy of the major allele of rs6565227 (T)	*ITGAM*	2.37 (1.23, 4.66)	0.006

## Supplementary Materials

The following are available online at http://www.mdpi.com/2073-4425/9/10/496/s1, Figure S1: Receiver operating characteristic (ROC) curve for LF model of SLE status including the recessive effect of the minor allele for all SNPS, gender, passive smoke exposure as a child and as an adult, and smoking status, Table S1: Genotype frequencies for each of the SNPs discussed in the Results, Discussion, and Conclusions.
