# Supplementary material for: An Analytic Approach Using Candidate Gene Selection and Logic Forest to Identify Gene by Environment Interactions (G × E) for Systemic Lupus Erythematosus in African Americans
Source: Genes (Basel). 2018 Oct 15;9(10):496. doi: 10.3390/genes9100496 (PMC6211136; doi:10.3390/genes9100496)
Supplement: Supplementary file 1 [file genes-09-00496-s001.zip › SupplementalMaterial.pdf]

Supplemental Material: An analytic approach using candidate gene selection and Logic Forest to identify gene by environment interactions (G×E) for systemic lupus erythematosus in African Americans.

Bethany J Wolf, Paula S Ramos, J Madison Hyer, Viswanathan Ramakrishnan, Gary S Gilkeson, Gary Hardiman, Paul J Nietert, and Diane L Kamen

October 11, 2018

Supplemental Table 1 – Genotype frequencies for each of the SNPs discussed in the Results, Discussion, and Conclusions.

**Gene**	**SNP**	**Genotype**	**Frequency (%)**
ITGAX	rs4632147	T/T	10.6
		T/C	42.6
		C/C	46.8
	rs58708589	C/C	15.2
		C/T	43.7
		T/T	41.1
IRF5	rs11761199	G/G	3.04
		G/A	21.7
		A/A	75.3
	rs11770589	A/A	22.0
		A/G	50.6
		G/G	27.4
ITGAM	rs4632147	T/T	10.6
		T/C	42.6
		C/C	46.8
	rs58708589	C/C	15.2
		C/T	43.7
		T/T	41.1

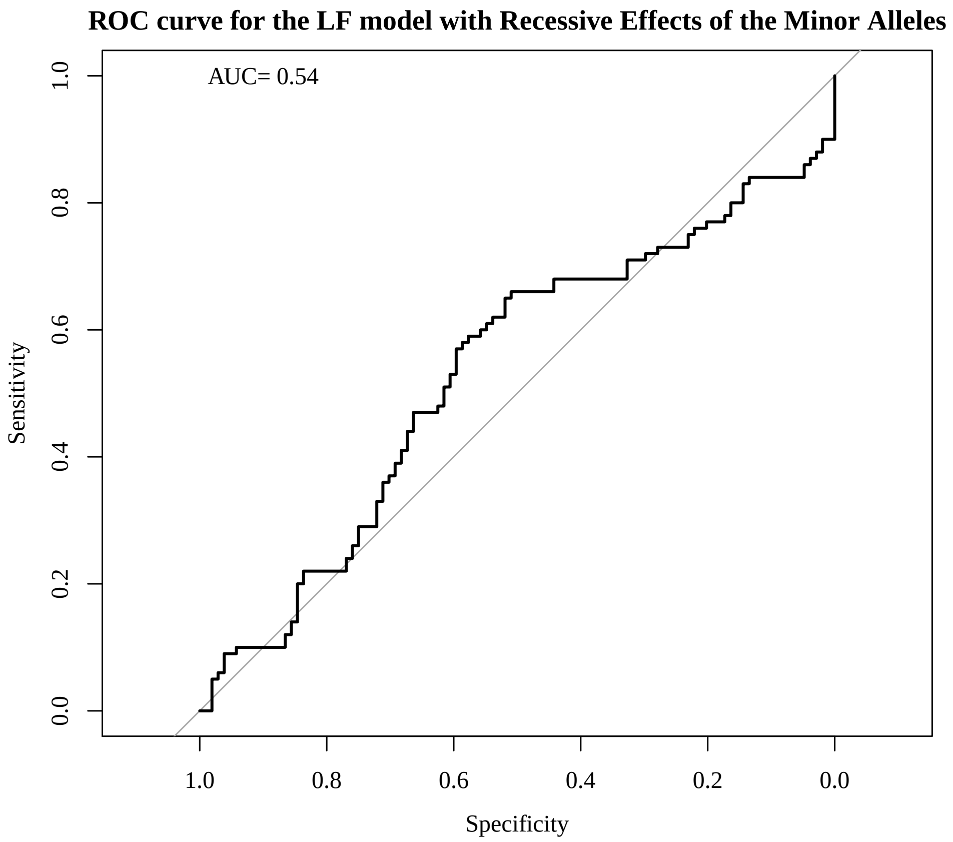

]

Supplemental Figure 1 – Receiver operating characteristic (ROC) curve for a Logic Forest model of SLE status including the recessive effect of the minor allele for all SNPS, gender, passive smoke exposure as a child and as an adult, and smoking status.
