# Supplementary figures and images for: An Analytic Approach Using Candidate Gene Selection and Logic Forest to Identify Gene by Environment Interactions (G × E) for Systemic Lupus Erythematosus in African Americans
Source: Genes (Basel). 2018 Oct 15;9(10):496. doi: 10.3390/genes9100496 (PMC6211136; doi:10.3390/genes9100496)

**ROC curve for the LF model with Recessive Effects of the Minor Alleles**

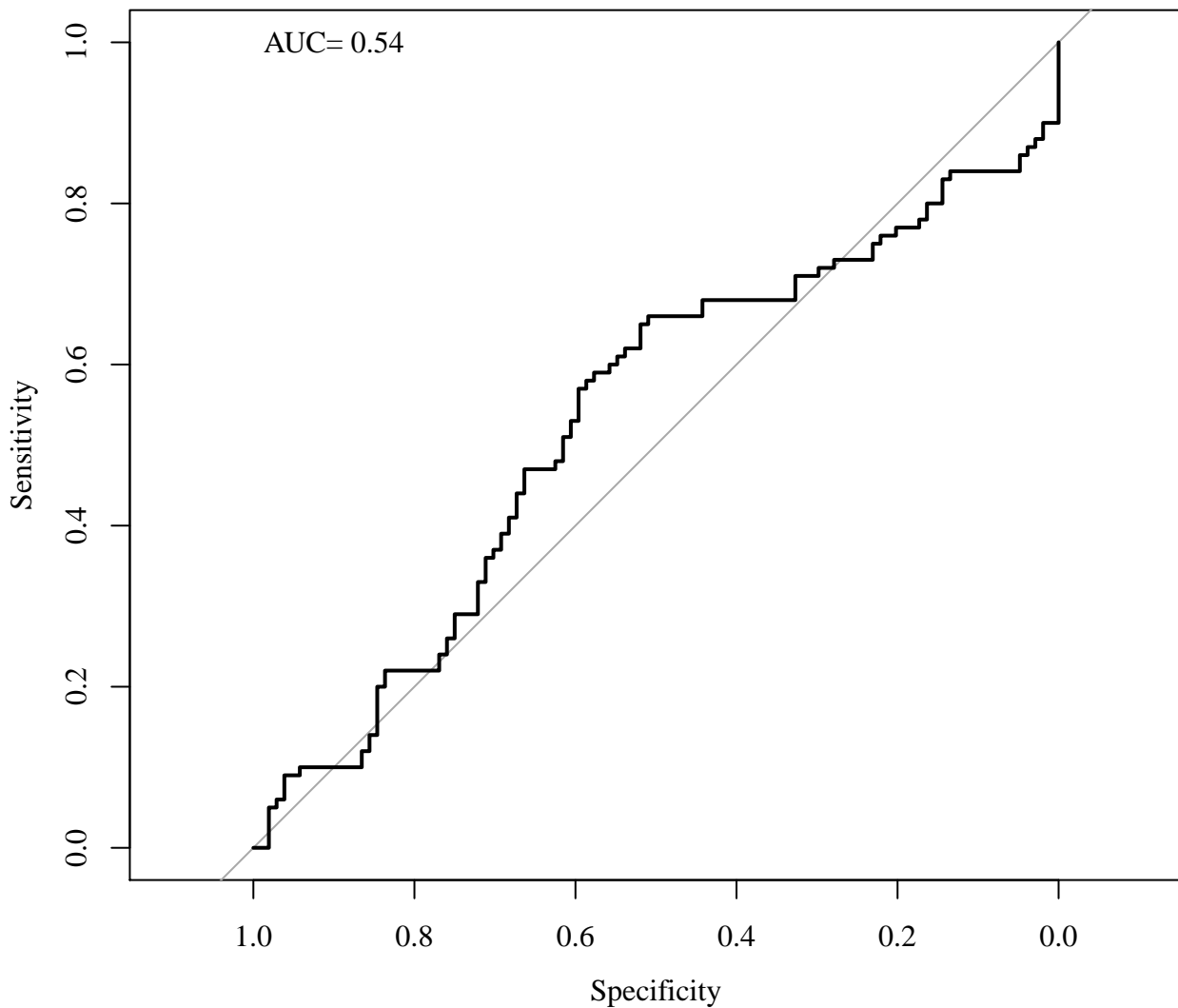

Supplement: Supplementary file 1 [file genes-09-00496-s001.zip › SuppFig1.pdf]
